# CHIPing away at immunity: the role of clonal hematopoiesis of indeterminate potential in bacterial pneumonia

**DOI:** 10.1172/JCI181064

**Published:** 2024-06-03

**Authors:** Elsa N. Bou Ghanem

**Affiliations:** Department of Microbiology and Immunology, Jacobs School of Medicine and Biomedical Sciences, University at Buffalo, Buffalo, New York, USA.

## Abstract

The occurrence of clonal hematopoiesis of indeterminate potential (CHIP), in which advantageous somatic mutations result in the clonal expansion of blood cells, increases with age, as do an increased risk of mortality and detrimental outcomes associated with CHIP. However, the role of CHIP in susceptibility to pulmonary infections, which also increase with age, is unclear. In this issue of the *JCI*, Quin and colleagues explored the role of CHIP in bacterial pneumonia. Using characterization of immune cells from human donors and mice lacking *tet methylcytosine dioxygenase 2 (Tet2)*, the authors mechanistically link myeloid immune cell dysfunction to CHIP-mediated risk of bacterial pneumonia. The findings suggest that CHIP drives inflammaging and immune senescence, and provide *Tet2* status in older adults as a potential prognostic tool for informing treatment options related to immune modulation.

## Pneumonia risk in older adults

Aging is a primary risk factor for pneumonia and is associated with adverse outcomes including death (1). Elucidating the mechanisms behind the age-driven susceptibility to lower respiratory tract infections is an area of active study that is needed to develop improved preventative and therapeutic approaches for enhancing longevity and quality of life as we age. Optimal host defenses balance rapid immune responses to control pathogen numbers followed by resolution of inflammation to prevent lung damage (2); however, aging is accompanied by persistent low-grade inflammation, known as inflammaging, as well as an overall decline in immune cell function, known as immunosenescence (2). These aberrant immune responses that accompany aging play a key role in susceptibility to pulmonary infections (2), though the etiologies behind these changes are incompletely defined.

## Clonal hematopoiesis of indeterminate potential

Aging is associated with an overall increase in somatic mutations in all tissues, including in hematopoietic stem cells in the bone marrow (3). An advantageous mutation results in clonal hematopoiesis and expansion of blood cell clones carrying the same mutation, leading to production of mutated immune cells (4). Clonal hematopoiesis of indeterminate potential (CHIP) refers to somatic mutations in genes involved in myeloid neoplasia where mutant clones and their variant alleles are found at a frequency of 2% or greater, but where no other criteria for frank hematological malignancy are met (4). Mutations leading to clonal hematopoiesis preferentially occur in certain genes, with approximately 67% occurring in the DNA methyltransferase 3 α (*DNMT3A*)*,* and tet methylcytosine dioxygenase 2 (*TET2*) genes, resulting in loss of enzymatic function (4). These enzymes have opposing roles: DNMT3A methylates while Tet2 demethylates DNA (4), and mutations in these genes result in myeloid skewing leading to production of monocytes and neutrophils with aberrant function (5, 6). The incidence of CHIP increases with age with an estimated occurrence in 10%–20% of older adults 65 years of age and above versus 1% in individuals under 40 years old (4). Several studies have found associations between CHIP and age-related diseases including cancer (7), cardiovascular diseases (8), impaired pulmonary function (9, 10), and increased mortality (11). Recent work has also linked CHIP with worse outcomes following SARS-CoV-2 infection (12). However, the link between CHIP-driven immune dysfunction and the age-associated inflammation and risk of bacterial pneumonia had not been explored.

## The role of CHIP in bacterial pneumonia

In this issue of the *JCI* (13), Quin and colleagues examined the role of CHIP in the age-associated increase in susceptibility to pulmonary infections. Using biobank data from a large observational cohort in the United Kingdom, they found that CHIP carriers had a 1.23-fold greater risk of pneumonia compared with noncarriers matched for covariates that impact infection susceptibility, including age and sex (14). They then assessed the association between CHIP and incidence of pneumonia caused by the bacteria *Streptococcus pneumoniae* (pneumococcus), which remains a leading cause of community-acquired pneumonias in older adults (1). CHIP carriers had a 2-fold greater incidence of pneumococcal pneumonia, but only in the presence of intact IL-6 signaling. This result fits with prior findings that had demonstrated a link between CHIP and inflammation in cardiovascular disease (15). These findings also shed light on the genetic factors that can govern heterogeneity of disease outcome following pneumococcal pneumonia (14).

**Figure 1:**
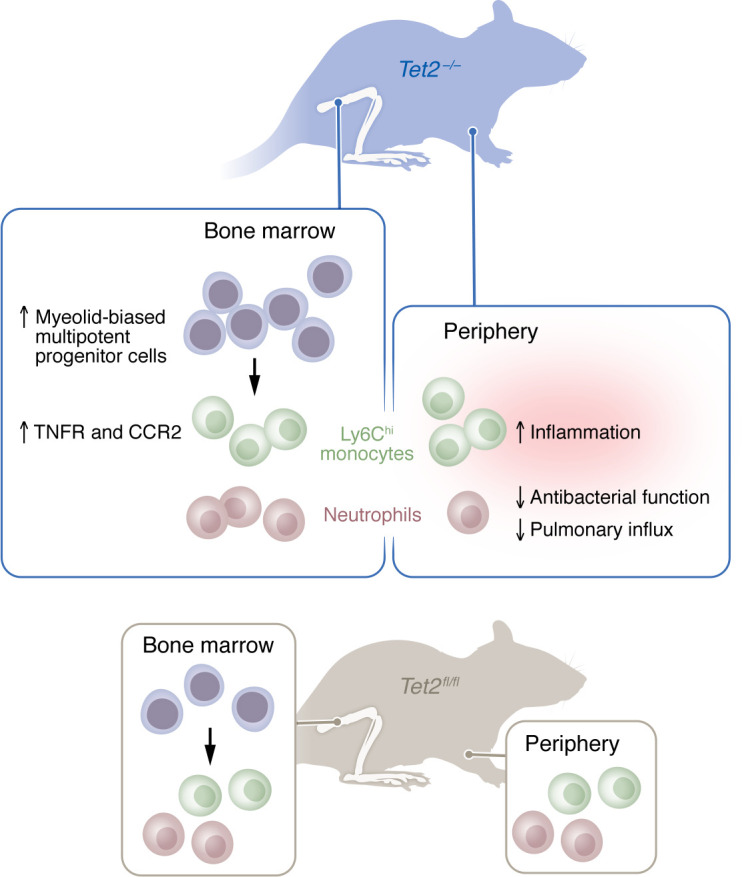
CHIP drives changes in immune cells. Compared with *Tet2^fl/fl^* mice, hematopoietic *Tet2*-knockout mice (*Tet2^–/–^*) display increased myelopoiesis in the bone marrow, increased inflammatory monocyte responses, and decreased neutrophil antimicrobial activity in the periphery.

In exploring mechanisms, the authors examined peripheral blood leukocytes in a cohort of older adults (over 68 years old) from Canada. CHIP in carriers (with 50% mutations in *TET2* and 50% in *DNMT3A*) contributed to changes in circulating cell populations, including myeloid skewing, expansion of monocytes, and neutrophils expressing lower levels of Fc-γ receptor, suggestive of lower function. The authors then directly assessed the role of Tet2 using murine models. Middle aged 8-to-10 month-old mice with *Tet2* deleted exclusively from hematopoietic cells (Tet2^–/–^) recapitulated the expansion of circulating myeloid cells seen in people with CHIP. The mutant mice also expanded circulating inflammatory Ly6C^hi^ monocytes that expressed higher levels of the proinflammatory cytokine TNF-α following LPS stimulation (13). In exploring why inflammatory monocytes were elevated in the circulation, the authors found that there was an increase in myeloid-biased multipotent progenitor cells in the bone marrow, demonstrating changes in myelopoiesis (Figure 1). They further found that Ly6C^hi^ monocytes in the bone marrow expressed elevated levels of CCR2 that are required for mobilization to the blood. Ly6C^hi^ monocytes also expressed higher levels of the TNF receptor. The authors had previously shown that TNF responsiveness provides a proliferative advantage in the bone marrow (16). Elevated TNF-α is known to impair immune cell function in aged hosts and contribute the enhanced susceptibility to pneumococcal pneumonia (17), and several aspects of immune impairment and pulmonary pathology seen in aging can be recapitulated by administration of TNF-α to young hosts (18). In fact, when Quin and authors tested the role of Tet2 in pneumococcal infection, middle aged mice with *Tet2* deleted from hematopoietic cells showed impaired bacterial clearance in the blood, enhanced systemic dissemination, and a reduction in overall survival following pulmonary challenge. The mutant mice exhibited overt pulmonary pathology but surprisingly lower levels of neutrophils in the pulmonary space (13).

Neutrophils are required for control of pneumococcal pneumonia, but their antimicrobial function and migration is impaired during host aging (2). Characterization of *Tet2* mutant neutrophils in Quin et al. (13) revealed many parallels to responses observed in older adults, including reduced chemotaxis toward stimuli, reduced migration to the lungs, and reduced phagocytosis, as well as intracellular killing of pneumococci (Figure 1) (2). Neutrophils from *Tet2-*mutant mice had lower expression of chemokine receptors as well as lower expression of genes in motility and migration pathways, explaining the compromised pulmonary influx (13). The impaired antimicrobial response of neutrophils was not specific to pneumococci alone, as exposure to *Staphylococcus aureus* failed to trigger effective production of neutrophil extracellular traps (NETs) in *Tet2-*mutant neutrophils (13). Since DNA methylation increases chromatin condensation, it is not surprising that loss of Tet2*,* which demethylates DNA, would result in smaller, more compact NETs. Impaired antimicrobial responses to other pathogens have been reported in *Tet2-*mutant neutrophils that exhibit blunted phagocytosis and clearance but enhanced inflammatory responses to *Candida albicans* (5). The findings in Quin et al. (13) show paradoxical enhanced inflammation but blunted antimicrobial capacity in the absence of *Tet2* from hematopoietic cells, similar to what is observed during aging (2).

## Implications and future directions

These findings by Quin and colleagues open several questions. The study identified dysfunction in the monocytic and neutrophilic compartment in the absence of *Tet2*; however, the mechanism driving increased susceptibility to bacterial pneumonia remains unclear. Is it the inflammatory monocytic response, the impaired neutrophilic bacterial clearance, or both? How about CHIP-driven changes in other cell types? Loss of function of Tet2 has been linked to an inflammatory phenotype in macrophages that contributes to cardiovascular disease (15). Since macrophages play an important role in the resolution of inflammation and return to homeostasis following lung injury (19), CHIP may also have a role in impaired pulmonary repair following infection. Another outstanding question relates to whether the mechanisms identified in Quin et al. (13) also hold true for other pneumonias, for example, of viral etiology. Prior studies have suggested a link between CHIP and elevated inflammation during SARS-CoV-2 infection (12), and aberrant neutrophilic responses play a detrimental role during viral pneumonias (20). The specific drivers of CHIP with age also remain unresolved. Inflammation has been shown to drive proliferation of *Tet2-*mutant clones (16), which, in turn, results in proinflammatory responses upon antigen exposure. Therefore, there could be a positive feedback loop between inflammaging and CHIP.

The study by Quin et al. also has potential clinical implications on disease management (13). As CHIP was associated with a higher risk of pneumonia as well as an exacerbated inflammatory response, it might serve as a prognostic tool for older adults that are carriers and inform treatment options, such as earlier interventions with immune modulating drugs. In summary, Quin and colleagues provide elegant mechanistic insight into CHIP-related susceptibilities (13). Beyond linking CHIP to bacterial pneumonia risk, they also connect *Tet2* mutations to myeloid dysfunction, including hyperinflammation and impaired antibacterial function, setting up CHIP as a potential driver of inflammaging and immune senescence.

## Version 1 06/03/2024

Electronic publication
